# Brain 5-HT_2A_ receptor binding and its neural network related to behavioral inhibition system

**DOI:** 10.1007/s11682-021-00609-2

**Published:** 2022-01-10

**Authors:** Kazuho Kojima, Shigeki Hirano, Yasuyuki Kimura, Chie Seki, Yoko Ikoma, Keisuke Takahata, Takehito Ito, Keita Yokokawa, Hiroki Hashimoto, Kazunori Kawamura, Ming-Rong Zhang, Hiroshi Ito, Makoto Higuchi, Satoshi Kuwabara, Tetsuya Suhara, Makiko Yamada

**Affiliations:** 1 Department of Neurology, Graduate School of Medicine, Chiba University, Chiba, 260-8670 Japan; 2 Department of Functional Brain Imaging, Institute for Quantum Medical Science, National Institutes for Quantum Science and Technology, Chiba, 263-8555 Japan; 3 Department of Clinical and Experimental Neuroimaging, Center for Development of Advanced Medicine for Dementia, National Center for Geriatrics and Gerontology, Obu, 474-8511 Japan; 4 Department of Molecular Imaging and Theranostics, Institute for Quantum Medical Science, National Institutes for Quantum Science and Technology, Chiba, 263-8555 Japan; 5 Department of Advanced Nuclear Medicine Sciences, Institute for Quantum Medical Science, National Institutes for Quantum Science and Technology, Chiba, 263-8555 Japan; 6 Department of Radiology and Nuclear Medicine, Fukushima Medical University, Fukushima, 960-1295 Japan; 7 Institute for Quantum Life Science, National Institutes for Quantum Science and Technology, 4-9-1 Anagawa, Inage-ku, Chiba, Chiba 263-8555 Japan

**Keywords:** Behavioral inhibition system, Serotonin system, Cingulate cortex, Positron emission tomography, Resting state functional magnetic resonance image

## Abstract

The tendency to avoid punishment, called behavioral inhibition system, is an essential aspect of motivational behavior. Behavioral inhibition system is related to negative affect, such as anxiety, depression and pain, but its neural basis has not yet been clarified. To clarify the association between individual variations in behavioral inhibition system and brain 5-HT_2A_ receptor availability and specify which brain networks were involved in healthy male subjects, using [^18^F]altanserin positron emission tomography and resting-state functional magnetic resonance imaging. Behavioral inhibition system score negatively correlated with 5-HT_2A_ receptor availability in anterior cingulate cortex. A statistical model indicated that the behavioral inhibition system score was associated with 5-HT_2A_ receptor availability, which was mediated by the functional connectivity between anterior cingulate cortex and left middle frontal gyrus, both of which involved in the cognitive control of negative information processing. Individuals with high behavioral inhibition system displays low 5-HT_2A_ receptor availability in anterior cingulate cortex and this cognitive control network links with prefrontal-cingulate integrity. These findings have implications for underlying the serotonergic basis of physiologies in aversion.

## Introduction

The fundamental features of complex behavior have long been discussed as being categorizable into the approach to rewards and the avoidance of punishments. These two systems can be applied to account for personality and motivation (Davidson, 1994; Gray, 1982; Higgins et al., 1994), positing that there are independent sensitivity in the respective systems. Gray provided a powerful theoretical framework that was rooted in behavioral psychology and neuroscience, called Reinforcement Sensitivity Theory (RST) (Gray, 1982). Gray proposes two systems together with an additional one: Behavioral Approach System (BAS), Behavioral Inhibition System (BIS), and Fight-Flight System (FFS) (see revised version of RST(Gray & McNaughton, 2000)). The BAS promotes behavior that leads to positive outcomes (reward and non-punishment) and is involved in the experience of positive emotions such as hope, elation, and happiness, while the BIS causes inhibition of behaviors that lead to negative outcomes (punishment and non-reward) and is involved in the experience of negative emotions such as fear, anxiety, frustration, and sadness. BAS corresponds to impulsivity, drug addiction, and attention deficit hyperactivity disorder; BIS is a self-reported sensitivity to punishment related to anxiety, depression, and pain, and FFS is fear at the psychological and psychiatric level (Bijttebier et al., 2009; Corr, 2002, 2004; Jensen et al., 2016).

In contrast to BAS, however, only a handful of studies have investigated the neural basis of BIS. The trait sensitivity to aversive events was associated with increased gray matter volume in amygdala and hippocampus (Barros-Loscertales et al., 2006; Cherbuin et al., 2008) and decreased volume in orbitofrontal cortex (OFC) and precuneus (Fuentes et al., 2012). BIS variability was also associated with individual differences in the neural activities of dorsal anterior cingulate cortex (ACC), OFC, striatum, amygdala, and hippocampus during anticipation of aversive events, such as monetary loss, measured by functional magnetic resonance imaging (fMRI) (Beaver et al., 2008; Kim et al., 2015; Simon et al., 2010). A resting-state fMRI (rs-fMRI) study similarly found that BIS correlated negatively with regional homogeneity in amygdala and hippocampus (Hahn et al., 2013).

Meanwhile, a number of neuroimaging studies have investigated the neural responses to aversive stimuli such as signal pain, punishment and monetary loss. The core regions of these aversive anticipations are found in ACC, anterior insula, OFC, and amygdala (De Martino et al., 2010; Eisenberger, 2012; Hayes & Northoff, 2011; Kringelbach & Rolls, 2004; Nitschke et al., 2006; Wrase et al., 2007). Congruent brain regions (ACC, OFC, amygdala) between aversive anticipation and individual variations in the sensitivity to aversive events leads to the notion that these regions are the hub for understanding the neural mechanisms of BIS.

According to Gray’s concept of BIS, harm avoidance is characterized by excessive anxiety and fear. Serotonin 2C(5-HT_2C_) receptors are linked to some of the adverse motivational effects corresponding to avoidance behaviors (Roberts et al., 2020), but 5-HT_2A_ receptors have also been reported to be intimately involved in the modulation of negative emotions, such as anxiety, depression, and pain (Baldwin & Rudge, 1995; Sommer, 2009). For instance, higher pessimistic behavior in depressive patients was related to higher frontal 5-HT_2A_ receptor binding as detected by positron emission tomography (PET) (Meyer et al., 2003). Harm avoidance and 5-HT_2A_ receptor availability showed a negative correlation in the prefrontal cortex and left parietal cortex (Moresco et al., 2002), a positive correlation in the dorsal prefrontal cortex (Baeken et al., 2014), or no significant regional correlation (Soloff et al., 2010). Although 5-HT_2A_ receptor agonists may experimentally increase impulsivity (Carli et al., 2006), the human [^18^F]altanserin PET study was unable to prove these relationships, in addition to a prior report (da Cunha-Bang et al., 2013; Frokjaer et al., 2008). Human PET studies have shown that 5-HT_2A_ receptors are numerous and widely distributed in cortical regions (Savli et al., 2012), and it remains unclear whether individual variations in 5-HT_2A_ receptor availability are involved in trait sensitivity to aversive events, that is, BIS.

The aim of this study was to elucidate the neural and molecular mechanisms associated with individual variations in BIS. In this regard, the relationships among BIS, the 5-HT_2A_ receptor availability using PET and the brain functional connectivity measured by rs-fMRI were investigated. We first conducted a PET imaging study to explore which brain regions of 5-HT_2A_ receptor availability correlated with BIS in healthy volunteers. Then, we analyzed rs-fMRI data to detect functional connectivity showing correlation with local 5-HT_2A_ receptor availability and BIS. Finally, mediation analysis was conducted to elucidate the relationships among BIS, functional connectivity and the 5-HT_2A_ receptor availability.

## Materials and methods

### Participants

Sixteen healthy right-handed male subjects (age: 23.3 ± 2.9 years, mean ± standard deviation) were recruited. Two subjects were excluded due to incomplete data collection, and the data of fourteen participants (23.4 ± 2.9 years) were analyzed. The demographic summary is shown in Table 1. All participants were free of current and past psychiatric or somatic disorders and had no history of drug abuse. Each participant completed psychological testing and underwent both rs-fMRI and PET scans. All participants provided written informed consent before participating in the study, which was approved by the Ethics and Radiation Safety Committee of the National Institute of Radiological Sciences in accordance with the ethical standards laid down in the 1964 Declaration of Helsinki and its later amendments.

**Table 1:** Demographic chart

Age (mean ± standard deviation)	23.4 ± 2.9
SP (median, [interquartile range])	61, [55 – 67.5]
SR	51, [44 – 59]
BHS	6, [4.5 – 10.5]
STAI	41, [34 – 54.5]

*SP* sensitivity to punishment, *SR* sensitivity to reward, *BHS* Beck Hopelessness Scale, *STAI* State-Trait Anxiety Inventory

### Psychological measurement

To test Gray’s original theory, Sensitivity to Punishment and Sensitivity to Reward Questionnaire (SPSRQ) was developed by Torrubia (Torrubia et al., 2001). This scale indicates good reliability and validity, and accurately expresses the essence of Gray’s theory (e.g., extraversion and neuroticism in expected directions). All participants completed the Japanese version of SPSRQ (Takahashi & Shigemasu, 2008). SPSRQ is a 48-item self-report measure that consists of two subscales, representing sensitivity to reward (SR) to measure impulsivity, i.e., BAS, and sensitivity to punishment (SP) to measure anxiety, i.e., BIS. Each item is scored on a 4-point Likert scale (1 = disagree, 4 = agree). SR and SP scores with higher scores indicating greater impulsivity and sensitivity to punishment, respectively. Participants also completed the Beck Hopelessness Scale (BHS(Beck et al., 1974)) and State-Trait Anxiety Inventory (STAI (Spielberger et al., 1983)) to measure the levels of depressive hopelessness and anxiety, respectively.

### PET acquisition and analysis

All subjects underwent a PET scan to measure regional 5-HT_2A_ receptor availability. Although [^18^F]altanserin is a reversible and selective antagonist of the rat 5-HT_2A_ receptor subtype (Riss et al., 2011), the changes in endogenous 5-HT binding do not directly influence the binding of [^18^F]altanserin (Kristiansen et al., 2005). A 90-min dynamic PET acquisition was performed after injection of [^18^F]altanserin (190 ± 5.4 MBq with molar activity of 167 ± 77 GBq/μmol). The scan protocol consisted of 33 frames (10 s × 6, 20 s × 3, 1 min × 6, 3 min × 4, and 5 min × 14 frames). All of the PET scans were performed on an Eminence SET-3000 GCT/X PET scanner (Shimadzu; Kyoto, Japan) with a head fixation device to minimize head movement. Each PET scan was preceded by a transmission scan for attenuation correction using a ^137^Cs source. All PET images were reconstructed with the filtered back-projection method (Gaussian filter, kernel 5 mm; reconstructed in-plane resolution was 7.5 mm in full width at half maximum; voxel size: 2 × 2 × 2.6 mm) corrected for attenuation, randoms and scatter.

During the scans, arterial blood samples were obtained manually 33 times after radioligand injection to obtain arterial input function (Ishii et al., 2017). Each blood sample was centrifuged to obtain plasma and blood cell fractions, and the concentrations of radioactivity in whole blood and plasma were measured (Ishii et al., 2017). The fractions of the parent compound and its radiometabolites in plasma were determined using high-performance liquid chromatography from 6 samples of each subject (Ishii et al., 2017).

All PET images were spatially normalized to the standard anatomic orientation. First, head motion during the scans was corrected on the emission images after correction of attenuation using µ-maps that were realigned to each frame of the emission images (Wardak et al., 2010). Second, T1-weighted MR images were coregistered to the corresponding mean PET images. Third, the MR images were spatially normalized and segmented into gray matter, white matter, and cerebrospinal fluid using SPM8 (Wellcome Institute of Neurology, University College of London, UK). Finally, all PET images were spatially normalized to the standard anatomic orientation (Montreal Neurological Institute (MNI) 152 standard space; Montreal Neurological Institute; Montreal, QC, Canada) based on the transformation of the MR images.

Because the Logan analysis provided a good compromise between validity, sensitivity, and reliability of implementation (Price et al., 2001), the PET data were analyzed by Logan graphical method (Logan et al., 1990), which was applied across the 12- to 90-min integration intervals, and regional total distribution volume (*V*_T_) values were obtained. We used the cerebellum as reference brain region and estimated the nondisplaceable distribution volume (*V*_ND_). 5-HT_2A_ receptor availability was determined as binding potential (*BP*_*P*_) that was derived from the equation: *BP*_*P*_ = *V*_T_–*V*_ND_ (Innis et al., 2007). All kinetic analyses were performed using PMOD (version 3.6, PMOD Technologies Ltd., Zurich, Switzerland).

### Region-of-interest analysis

ACC, OFC and amygdala, which are involved in the sensitivity to aversive events (Barros-Loscertales et al., 2006; Beaver et al., 2008; Cherbuin et al., 2008; Eisenberger, 2012; Fuentes et al., 2012), were applied to ROI analyses. These brain regions were extracted by the Harvard–Oxford atlas using the CONN toolbox (version 17e, http://www.nitrc.org/projects/conn), averaged over right and left. Subsequently, ACC was divided into four segregated subregions, namely, subgenual ACC (sgACC), pregenual ACC (pgACC), anterior midcingulate cortex (aMCC) and posterior midcingulate cortex (pMCC) (Yeung et al., 2004). Subgenual ACC was substituted by subcallosal cortex in the atlas due to small volume (0.056 mm^3^). The volumes of each ACC subregion were 9.2 mm^3^ in subcallosal cortex, 6.5 mm^3^ in pgACC, 7.6 mm^3^ in aMCC, and 6.7 mm^3^ in pMCC. The volumes of the OFC and amygdala were 25.3 mm^3^ and 5.4 mm^3^, respectively.

### Resting-state fMRI acquisition and analysis

Each subject underwent a 6.8-min rs-fMRI scan, performed with a Magnetom Verio 3.0 T MRI scanner (Siemens, Erlangen, Germany) equipped with a 32-channel head coil. During scanning, subjects were instructed to relax with their eyes open while gazing at a fixation cross. A single session acquired 3.8-mm thick, no gap, interleaved axial 33 slices (in-plane resolution: 3.75 × 3.75 mm) with a 30-degree angle relative to the AC-PC axis, using a T2*-sensitive single-shot EPI sequence with the following parameters: TR = 2000 ms, TE = 25 ms, flip angle = 90 degrees, matrix = 64 × 64. A high-resolution T1-weighted anatomical image using a magnetization prepared rapid acquisition gradient echo (MPRAGE) sequence (176 sagittal slices, resolution = 0.49 × 0.49 × 1.00 mm, no gap, TR = 2300 ms, TE = 1.95 ms, flip angle = 9 degrees, matrix = 512 × 512) was acquired for anatomical reference.

Data processing was performed using the CONN toolbox and SPM12 (Wellcome Institute of Neurology, University College of London, UK) working on Matlab version 8.4 (MathWorks, MA, USA). The first four volumes were discarded from analysis to account for magnetization saturation effects. Preprocessing comprised: 1) realignment and unwarping, 2) slice timing correction, 3) segmentation and normalization, 4) smoothing with a Gaussian kernel of 4 mm. To eliminate correlations caused by head motion and artifacts, we identified outlier time points in the motion parameters and global signal intensity using Artifact Detection Tools (ART), which includes the CONN toolbox. For each subject, we treated images as outliers if composite movement from a preceding image exceeded 0.2 mm, or if the global mean intensity was over 3 SDs from the mean image intensity for the entire resting scan. Based on the previous report(Yan et al., 2013), after removing outlier images, one subject whose total scan time of less than 3 min was excluded from the subsequent analyses.

After preprocessing, we conducted de-noising as follows: 1) linear regression of noise sources from white matter and cerebrospinal fluid by CompCor (component-based noise correction method) and from outliers by ART and from Friston 24 head motion parameter, 2) band-pass filtering of 0.009 – 0.1 Hz was used to pass the low frequency fluctuations of interest, 3) quadratic trends were removed. Global signal regression was not used to avoid potential false anticorrelations.

### Image analyses

#### Association between SP and 5-HT_2A_ receptor availability

To test for the contribution of 5-HT_2A_ receptor availability to SP, we first conducted Spearman’s rank test between SP and the *BP*_*P*_ value of each ROI using GraphPad Prism (version 7, GraphPad Software, CA, USA). P-value less than 0.05 with false discovery rate (FDR) correction for multiple comparisons was considered significant.

#### Relationship between 5-HT_2A_ receptor availability and functional connectivity

Regions of 5HT_2A_ receptor availability that correlated with SP were subsequently investigated by seed-based functional connectivity analysis modeling the *BP*_*P*_ values in the analogous regions, utilizing the CONN toolbox. This procedure may explore the functional connectivities that correlate with 5-HT_2A_ receptor availability of SP-related ROIs. The threshold was defined as a cluster-level threshold of *p* < 0.05, FDR-corrected with voxel-level threshold of *p* < 0.001, uncorrected for multiple comparisons. All reported coordinates were of MNI standard space.

#### Functional connectivity related to SP

The correlation coefficient for each specified functional connectivity was extracted. Spearman’s rank test was performed between each extracted correlation coefficient and SP. P-value less than 0.05 was considered significant.

#### Mediation analysis

Finally, for each functional connectivity that was significantly related to both 5-HT_2A_ receptor availability and SP, we performed mediation analysis to test whether functional connectivity might be involved in the link between SP and 5-HT_2A_ receptor availability. The correlation coefficient of functional connectivity was included as a mediator. INDIRECT macro (Preacher & Hayes, 2008) with SPSS (version 24, IBM, NY, USA) was used. Bias-corrected and accelerated 95% confidence intervals based on 10,000 bootstrap sampling were used to assess significance.

## Results

### Behavioral findings

The median SP and SR scores were 61 (interquartile range, 55–67.5) and 51 (interquartile range, 44–59), respectively (Table 1). The median score of BHS was 6 (interquartile range, 4.5–10.5) and STAI was 41 (interquartile range, 34–54.5). The SP scores correlated positively with the BHS (r_s_ = 0.86, *p* < 0.0005, Spearman’s rank test) and at a marginally significant level with STAI (r_s_ = 0.52, *p* = 0.07).

### Regional 5-HT_2A_ receptor availability measured by [^18^F]altanserin PET

**Fig. 1:**
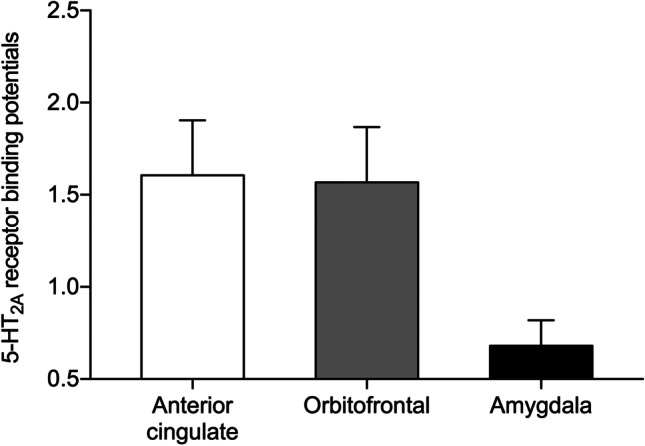
[^18^F]altanserin binding potentials of the limbic system. Bar graphs represent mean ± standard deviation

Figure 1 shows the 5-HT_2A_ receptor availability values (*BP*_*P*_) in ACC, OFC and amygdala which were selected from the premise that these regions were associated with aversive anticipation. Higher *BP*_*P*_ was measured in the ACC and OFC, while low *BP*_*P*_ was observed in the amygdala. The *BP*_*P*_ values in ACC (1.535 [1.376 to 1.773]), OFC (1.503 [1.41 to 1.633]) and amygdala (0.676 [0.581 to 0.762]) were comparable to that of healthy subjects in previous report (Savli et al., 2012).

### Association between behavioral inhibition system and 5-HT_2A_ receptor availability

The SP score was negatively correlated with the *BP*_*P*_ value of ACC (r_s_ = -0.66, *p* = 0.016, Spearman’s rank test with FDR correction). No correlations were found in OFC or amygdala (r_s_ = -0.47 and r_s_ = -0.57, respectively, both *p* > 0.05 with FDR correction). There was no significant correlation between the SR score and *BP*_*P*_ values (ACC, r_s_ = 0.28, *p* = 0.35; OFC, r_s_ = 0.02, *p* = 0.96; amygdala, r_s_ = 0.11, *p* = 0.73).

**Fig. 2:**
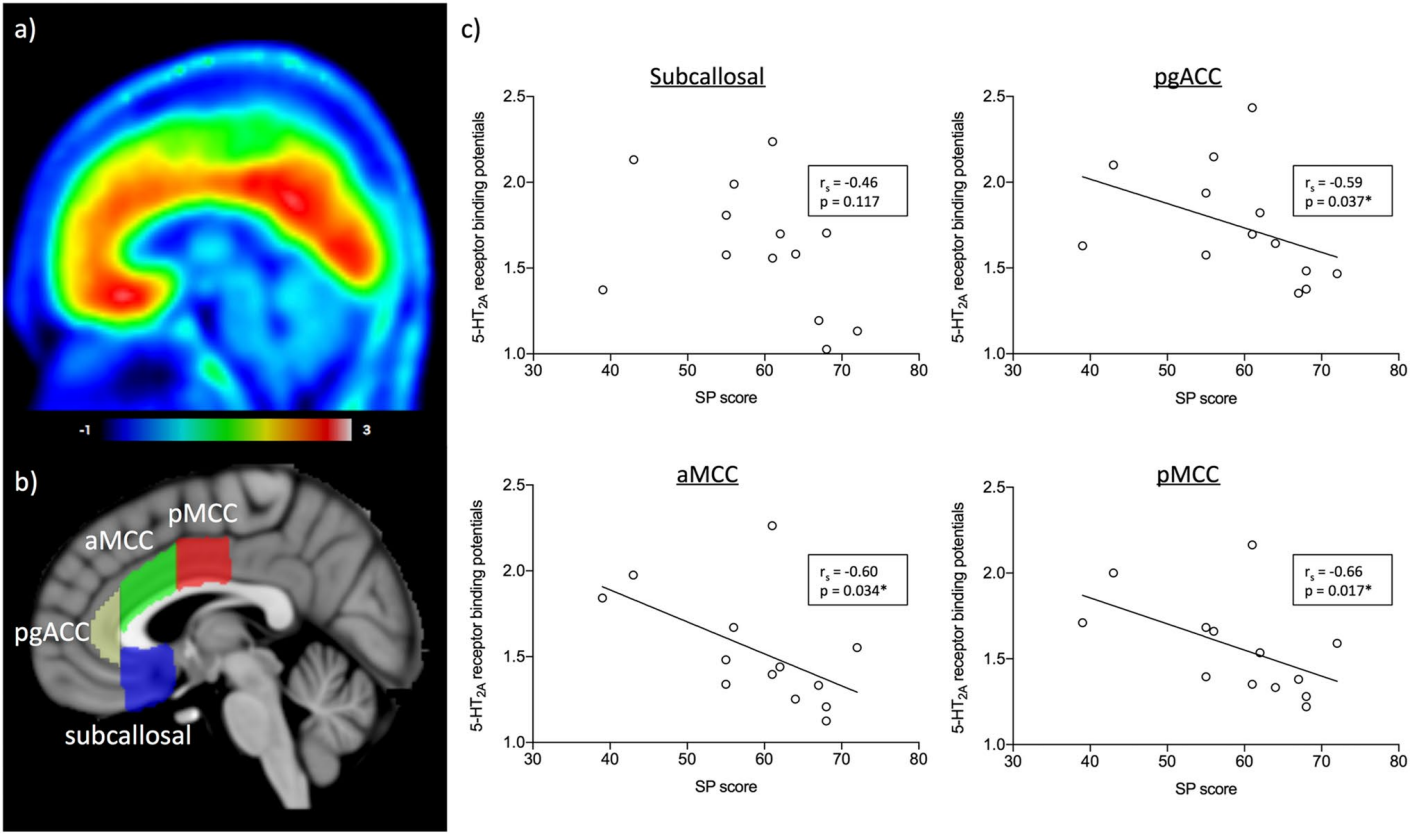
**a**) Mean parametric image of 5-HT_2A_ receptor binding of [^18^F]altanserin PET, shown in sagittal view. **b**) Subdivisions of ACC, overlaid on sagittal T1 MRI template. **c**) Plot graph of sensitivity to punishment (SP) score and regional 5-HT_2A_ receptor binding potentials (*BP*_*P*_). SP was negatively correlated with 5-HT_2A_ receptor *BP*_*P*_ in pgACC, aMCC and pMCC, whereas no such association was detected in subcallosal region (*false discovery rate corrected *p* < 0.05). Spearman ‘s rank test was used. pgACC, pregenual anterior cingulate cortex; aMCC, anterior midcingulate cortex; pMCC, posterior midcingulate cortex

We further examined the correlations between SP and the *BP*_*P*_ values in the functional subdivisions of ACC. There was no difference in the *BP*_*P*_ values among subdivisions of ACC (F(3, 48) = 1.06, *p* = 0.375, One-way ANOVA). SP scores correlated negatively with the *BP*_*P*_ values in pgACC, aMCC and pMCC but not in subcallosal cortex (pgACC, r_s_ = -0.59, *p* = 0.037; aMCC, r_s_ = -0.60, *p* = 0.034; pMCC, r_s_ = -0.66, *p* = 0.017; subcallosal cortex, r_s_ = -0.46, *p* = 0.117, Spearman’s rank test with FDR correction; Fig. 2).

### Association between 5-HT_2A_ receptor availability and functional connectivity

**Fig. 3:**
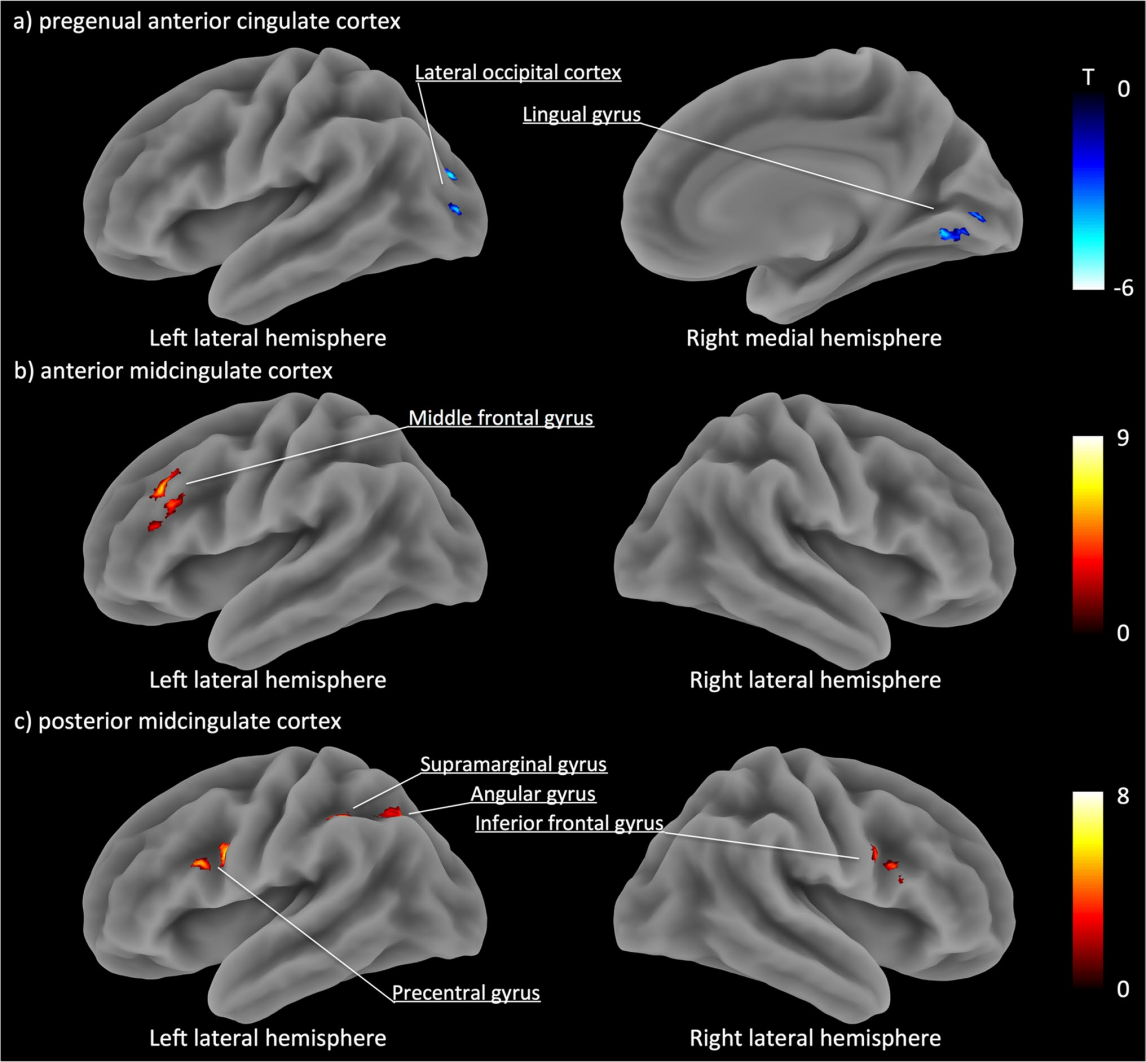
Surface rendered images of functional connectivity associated with 5-HT_2A_ receptor binding potentials in each anterior cingulate cortex subregion. **a**) 5-HT_2A_ receptor binding potentials in the pgACC were negatively correlated with the functional connectivity of the pregenual anterior cingulate cortex. **b**) 5-HT_2A_ receptor binding potentials in the anterior midcingulate cortex were positively correlated with the functional connectivity of the anterior midcingulate cortex. **c**) 5-HT_2A_ receptor binding potentials in the posterior midcingulate cortex were positively correlated with the functional connectivity of the posterior midcingulate cortex. Shown clusters remained after a threshold of cluster-level *p* < 0.05 false discovery rate corrected and voxel-level *p* < 0.001 uncorrected for multiple comparisons. Clusters were surface-rendered onto a brain template. Color bar represents T-value; negative correlations as blue-purple, positive correlations as red-yellow

Seed-based functional connectivity analyses were performed for above three ACC subregions (Fig. 3, Table 2) to explore functional connectivity that correlated with the local *BP*_*P*_ value. The *BP*_*P*_ values in pgACC were negatively correlated with the functional connectivity between pgACC and clusters in left lateral occipital cortex and right lingual gyrus (Fig. 3a). The *BP*_*P*_ values in aMCC were positively correlated with the functional connectivity between aMCC and left middle frontal gyrus (MFG) (Fig. 3b). The *BP*_*P*_ values in pMCC were positively correlated with the functional connectivity between pMCC and clusters in right inferior frontal gyrus, left precentral gyrus, left supramarginal gyrus and left angular gyrus (Fig. 3c).

**Table 2:** Coordinates of functional connectivity that correlated with 5-HT_2A_ receptor binding potentials in each anterior cingulate cortex subregion

Brain Region	Extent	T-value	MNI Coordinates		
			x	y	z
ROI: pregenual anterior cingulate cortex					
Left lateral occipital cortex	54	-5.70	-22	-80	14
Right lingual gyrus	33	-5.77	14	-72	-2
ROI: anterior midcingulate cortex					
Left middle frontal gyrus	91	8.35	-36	28	26
ROI: posterior midcingulate cortex					
Left precentral gyrus	119	7.27	-50	2	26
Left supramarginal gyrus	46	7.23	-42	-44	44
Left angular gyrus	67	6.84	-28	-58	34
Right inferior frontal gyrus	50	5.72	52	18	28

A cluster-level threshold of *p* < 0.05 false discovery rate corrected with voxel-level threshold of *p* < 0.001 uncorrected

*ROI* Region-of-interest, *MNI* Montreal Neurological Institute

### Functional connectivity related to behavioral inhibition system

Correlation analyses between these specific functional connectivities and SP were carried out. SP was negatively correlated with the functional connectivity between aMCC and left MFG (r_s_ = -0.67, *p* = 0.014, Spearman’s rank test; Table 3).

**Table 3:** Correlations between sensitivity to punishment score and functional connectivity of each subregion of anterior cingulate cortex

	r_s_	*p* value
ROI: pregenual anterior cingulate cortex (pgACC)		
pgACC—left lateral occipital cortex	0.52	0.069
pgACC—right lingual gyrus	0.44	0.135
ROI: anterior midcingulate cortex (aMCC)		
aMCC—left middle frontal gyrus	-0.67	0.014*
ROI: posterior mid cingulate cortex (pMCC)		
pMCC—left precentral gyrus	-0.46	0.112
pMCC—left angular gyrus	-0.39	0.189
pMCC—right inferior frontal gyrus	-0.35	0.243
pMCC—left supramarginal gyrus	-0.41	0.166

*r*_s_ Spearmann's rank correlation coefficient, *ROI* Region-of-interest

^*^p < 0.05

### Mediation analysis

Finally, we examined whether functional connectivity between aMCC and left MFC serve as a potential mediator of the link between SP scores and 5-HT_2A_ receptor availability. We tested two possible models: 1) 5-HT_2A_ receptor availability affects functional connectivity, which in turn affects SP; 2) SP affects functional connectivity, which in turn affects 5-HT_2A_ receptor availability. Mediation analyses supported the latter model, indicating that the total indirect effect of SP scores on the *BP*_*P*_ values via functional connectivity was significant (Bias-corrected and accelerated 95% confidence intervals: -0.044 to -0.007; *p* < 0.05). In sum, these results imply that the functional connectivity between aMCC and left MFG serves as an important role in linking BIS with the 5-HT_2A_ receptor availability.

## Discussion

This study investigated whether the individual variations in 5-HT_2A_ receptor availability contributes to BIS and which brain networks were specifically involved. BIS correlated negatively with 5-HT_2A_ receptor availability in ACC, and the association between BIS and 5-HT_2A_ receptor availability was accounted for by the functional connectivity between aMCC and left MFG.

Our findings indicate the role of 5-HT_2A_ receptor-mediated serotonergic neurotransmission in ACC, as was previously linked with BIS personality and aversive anticipation. Specifically, high BIS individuals showed reduced levels of serotonin 5-HT_2A_ receptor availability in ACC. The 5-HT_2A_ receptor is known to be related to psychiatric symptoms, such as anxiety and depression, as well as hallucinations in schizophrenia (Quednow et al., 2009) and Parkinson’s disease (Ballanger et al., 2010). In recent genetic studies, polymorphism of the 5-HT_2A_ gene has been frequently reported in depression and schizophrenia (Gu et al., 2013; Tan et al., 2014; Zhao et al., 2014). For example, single nucleotide polymorphism of 5-HT_2A_ gene was associated with pathological gambling and suicide in depressed patients (Arias et al., 2001; Wilson et al., 2013). Furthermore, in PET studies, while suicide victims had a high density of 5-HT_2A_ receptors in prefrontal cortex (Du et al., 2001), treatment-resistant depressed patients displayed lower 5-HT_2A_ receptor binding in dorsal prefrontal cortex and ACC (Baeken et al., 2012). These contradictory findings represent the activation and the inhibition of impulsivity (Fineberg et al., 2010), and the current finding supports to the latter. Animal experiments have also shown that blockage of the 5-HT_2A_ receptor in medial prefrontal cortex suppressed impulsive behavior (Fink et al., 2015); human experiments have shown that 5-HT_2A_ agonists facilitate punishment learning (Kanen et al., 2021). A recent review suggested that 5-HT_2A_ signaling is associated with cognitive flexibility (Carhart-Harris & Nutt, 2017). Although the causal relationship between 5-HT_2A_ receptor availability and BIS still needs to be verified, the present results of mediation analyses indicate that a high level of BIS, which positively correlates with depressive hopelessness, causes low 5-HT_2A_ receptor availability in aMCC via the functional connectivity of aMCC. This may reflect the inhibitory control and cognitive flexibility associated with some aspects of depressive symptoms, which may lead to downregulation of the 5-HT_2A_ receptor.

This study newly identified the functional connectivity associated with 5-HT_2A_ receptor availability in ACC subregions (pgACC, aMCC, pMCC), and the mediation test revealed that aMCC-MFG functional connectivity contributed to the link between BIS and 5-HT_2A_ receptor availability. ACC has been consistently linked with cognitive function, emotion processing, and the autonomic nervous system (Bush et al., 2000; Critchley et al., 2001), which is divided into 4 subregions (Vogt et al., 2003). The pgACC, the ventral part of ACC, is involved in assessing emotional and motivational information. The pMCC and aMCC are part of the dorsal ACC, mainly involved in cognitive controls. The negative correlation between the *BP*_*P*_ of the pgACC and the functional connectivity between the pgACC and left lateral occipital cortex/right lingual gyrus might be associated with the psychedelic state, although it is not possible to draw any conclusions from the current results. Lysergic acid diethylamide (LSD), a non-selective 5-HT_2A_ receptor agonist and well-known hallucinogenic agent, was administered to healthy subjects who underwent fMRI scanning, which revealed an association with the activation of the primary visual cortex, which was correlated with the degree of visual hallucination, represented as a ‘psychedelic state’ (Carhart-Harris et al., 2016).

The aMCC represents a hub where information about punishment and negative feedback, such as pain, is monitored, triggering control signals and/or selective attention generated in dorsolateral prefrontal cortex (DLPFC) (MacDonald et al., 2000; Miller & Cohen, 2001; Shackman et al., 2011; Walsh et al., 2011; Yeung et al., 2004). Other studies have also shown that aMCC is anatomically connected with DLPFC in monkeys (Morecraft & Tanji, 2009), and that the functional connectivity between these two regions is correlated with working memory demand according to task-based fMRI (Osaka et al., 2004). A recent study with multi-voxel pattern analysis supports the fact that the middle frontal gyri appear to be primarily predictive of the subjective experience of fear (Taschereau-Dumouchel et al., 2020). Consistent with these previous studies, our finding of the functional connectivity between aMCC and left MFG, a part of DLPFC, possibly reflects the inhibitory control and cognitive flexibility associated with negative information processing, and in particular, serves a mechanistic role in linking BIS and 5-HT_2A_ receptor–mediated serotonergic neurotransmission.

It is puzzling that the direction of the path was found to be from the psychological trait to the molecular system, not vice versa. The mediation test taps on the mathematical linkage rather than on a biological one. 5-HT_2A_ receptor–mediated stimulation by physiological and acute or chronic pharmacological manner may exhibit differently in the brain function. To further clarify this question, acute and chronic 5-HT_2A_ receptor intervention may alter both BIS and the functional connectivity between aMCC and left MFG, which shall be left for the future investigations.

There are several limitations to this study. The first is that our sample size is comparatively small. Although we have set a stringent statistical threshold, future study with a larger sample size will be required to replicate the current findings. Second, females were not included in the present study. As estrogen promotes 5-HT synthesis and menstrual cycle, it influences 5-HT_2A_ receptor binding in women (Wihlbäck et al., 2004), thereby we exclusively included male subjects in the current study. Considering that emotional reactions differ between genders, it may be interesting to explore the similarities and differences between male and female subjects in the future. In addition, the enrolled subjects were all Japanese. Previous behavioral studies have indicated that Japanese are motivated more by negative feedbacks than by positive ones (Diener et al., 2003; Heine et al., 2001); thus, our results might be biased in this regard. Lastly, functional connectivity only accounts for a linear association between two brain regions. Whole brain networks and anatomical connectivities were not examined in the present study and should be addressed in the future studies.

## Conclusions

In summary, this multimodal neuroimaging study provides novel evidence of the relationship between the behavioral inhibition and the 5-HT_2A_ receptor–mediated serotonergic function, which is mediated by the functional connectivity between aMCC and left MFG, known as a cognitive control network. The link obtained in the current study may be tested by interventional studies using drugs which modulate 5-HT_2A_ receptor function to elucidate biological causal relationships. From the basis from the current findings, the symptoms related with behavioral inhibition of patients with anxiety, depression, and pain disorder may benefit from medications associated with 5-HT_2A_ receptor function.

## Data Availability

Not applicable.
